# The Effects of Rilmenidine and Perindopril on Arousal Blood Pressure during 24 Hour Recordings in SHR

**DOI:** 10.1371/journal.pone.0168425

**Published:** 2016-12-21

**Authors:** Kyungjoon Lim, Kristy L. Jackson, Sandra L. Burke, Geoffrey A. Head

**Affiliations:** 1 Neuropharmacology Laboratory, Baker IDI Heart and Diabetes Institute, Melbourne, Victoria, Australia; 2 Department of Pharmacology, Monash University, Clayton, Victoria, Australia; Universidad de Buenos Aires, ARGENTINA

## Abstract

The surge in arterial pressure during arousal in the waking period is thought to be largely due to activation of the sympathetic nervous system. In this study we compared in SHR the effects of chronic administration of the centrally acting sympatholytic agent rilmenidine with an angiotensin converting enzyme inhibitor perindopril on the rate of rise and power of the surge in mean arterial pressure (MAP) that occurs with arousal associated with the onset of night. Recordings were made using radiotelemetry in 17 adult SHR before and after treatment with rilmenidine (2mg/kg/day), perindopril (1mg/kg/day) or vehicle in the drinking water for 2 weeks. Rilmenidine reduced MAP by 7.2 ± 1.7mmHg while perindopril reduced MAP by 19 ± 3mmHg. Double logistic curve fit analysis showed that the rate and power of increase in systolic pressure during the transition from light to dark was reduced by 50% and 65%, respectively, but had no effect on diastolic pressure. Rilmenidine also reduced blood pressure variability in the autonomic frequency in the active period as assessed by spectral analysis which is consistent with reduction in sympathetic nervous system activity. Perindopril had no effect on the rate or power of the arousal surge in either systolic or diastolic pressure. These results suggest that the arousal induced surge in blood pressure can largely be reduced by an antihypertensive agent that inhibits the sympathetic nervous system and that angiotensin converting enzyme inhibition, while effective in reducing blood pressure, does not alter the rate or power of the surge associated with arousal.

## Introduction

Hypertension is an important risk factor for predicting cardiovascular disease but it is the morning period that is the period of greatest risk of stroke and myocardial infarcts [1–6]. During the morning period there is a gradual increase in blood pressure (BP) associated with the normal circadian pattern in humans as BP moves towards its higher daytime level. This process is not a sudden jump but takes several hours. While methods for analysis of diurnal changes in cardiovascular variables have not easily determined the rate of change in BP during different periods of the day, we have devised a new mathematical analysis which can estimate the rate of change in BP and heart rate (HR) during the transitions between sleep and awake. We have shown that hypertensive humans [7] and rats [8] have a greater rate of rise in BP during the period of arousal from sleep compared to normotensives. We have also shown that this greater rate of rise in BP is a significant and independent risk factor in humans [9] and is related to the activation of the sympathetic nervous system [10]. Earlier studies compared the frequency of cardiac synchronised sympathetic bursts in the perineal nerve and did not show a difference between the morning and evening period, suggesting that there was no difference between sympathetic activity in these periods [11]. However, it is the amplitude of the burst that we found is related to the morning surge in blood pressure and not the frequency of firing [10]. Importantly the amplitude of the sympathetic burst is also elevated in conditions such as experimental hypertension induced by angiotensin infusion and hypoxia [12]. The amplitude represents the activity of only active fibres which under normal conditions is a minority with the majority being “silent” or “inactive”. An increase in burst amplitude therefore suggests that previously silent fibres are being recruited to become active. We recently confirmed that individual sympathetic units did not increase firing rate in hypertension [13]. Taken together, these findings suggest that the morning surge in blood pressure that occurs during arousal is characterised by activation of new sympathetic fibres.

While the rate of rise in BP is clearly important, the magnitude of the rise also hasconsiderable influence on the impact of the rise in pressure. Indeed most measures such as that developed by Kario and colleagues have used an estimate of the morning change in BP within a specified period of waking [14]. Termed the morning BP surge (MBPS), this measure has been extensively used in the literature. We have recently developed a novel measure of the morning surge in BP which we have termed the “BP_Power_” which is the product of the rate and the amplitude of the BP morning surge [1]. BP power is 2.5 fold greater in hypertensive subjects than matched normotensive patients [1] and may therefore represent more effectively the impact of the morning surge [1]. We recently compared the rate of rise, BPpower and MBPS with activation of the sympathetic nervous system in 35 patients and found that the sympathetic burst amplitude was most related to the BP_Power_ and rate of rise but not at all the MBPS [10]. Thus we hypothesise that the morning BP_Power_ would be most susceptible to attenuation with pharmacological agents that target the sympathetic nervous system such as centrally acting antihypertensive agents. We have extensive experience with rilmenidine and moxonidine which are second generation agents of this class that have mixed actions on α_2_-adrenoceptrors and imidazoline receptors [15]. The principle antihypertensive effects of rilmenidine and moxonidine are through inhibition of sympathetic activity [16] and this involves mainly activation of imidazoline receptors in the rostral ventrolateral medulla [17–19]. Rilmenidine is also known to facilitate the cardiac baroreflex through greater vagal activity but only during the light inactive period in mice [20]. We have also observed that rilmenidine reduced the rate of rise in BP that occurs at the onset of darkness in hypertensive mice [20]. However, we do not know the effect of rilmenidine on the arousal BP_Power_ nor whether this is simply due to a reduction in BP itself.

Hence, the aim of the present study was to apply the new logistic curve fitting procedure to BP recordings from rats in order to determine the effects of chronic administration of the central sympatholytic agent rilmenidine on telemetry recordings of BP, HR and locomotor activity in spontaneously hypertensive rats (SHR). In particular we tested the hypothesis that the sympatholytic agent rilmenidine would attenuate the rate of rise in BP and the BP_Power_ during night onset arousal. We also compared the findings with an angiotensin converting enzyme inhibitor perindopril to determine the effect of reducing BP but not affecting the SNS.

## Materials and Methods

### Animals, surgical procedures

All procedures were approved by the Alfred Hospital/Baker Heart & Diabetes Institute Animal Ethics Committee and were carried out according to guidelines set by the National Health and Medical Research Council of Australia. Eighteen male 12–14 week old SHR were obtained from the Baker Heart & Diabetes Institute, weighing 341 ± 11g at the onset of the experiments.

### Telemetry probe implantation

In an initial operation, the rats were instrumented with a radio-telemetry probe (Data Sciences, MA, USA) as previously described [21]. The rats were anesthetized with halothane delivered by a mask from an open circuit vapourizer (Goldman) and the abdominal aorta exposed through a midline incision. The blood pressure sensor (model TA11PA-C40) was inserted into the aorta close to the femoral bifurcation and fixed with a drop of tissue glue. The body of the transmitter was sutured to the inside of the abdominal muscle wall. All incisions were then sutured and the rat was given carprofen for analgesia (5 mg/kg, Pfizer, NSW, Australia) and allowed to recover from anesthesia in a warm box. After surgery, the rats were housed singly with free access to tap water and standard rat pellet chow.

### Radio telemetry recording method

The radio transmitter, once implanted and turned on by a magnetic switch, provided a continuous measure of the arterial pressure wave form as well as a measure of locomotor activity [21]. The AM radio signals were an encoded pulse position modulated serial bit stream, collected by the receiver (model RPC-1, Data Sciences International) placed underneath the animal’s cage. The signals from the receiver were passed to an analogue converter (model RP11A, Data Sciences International). Ambient barometric pressure was also measured (APR-1, Data Sciences International) and subtracted from the telemetered pressure by the data collection system in order to compensate for changes in atmospheric pressure. The analogue voltage signal was then converted by an analogue to digital acquisition card (PC plus, National Instruments, Austin Texas, USA) using software (Universal acquisition) written in Labview (National Instruments).

### Experimental protocol

After 1–2 weeks of post-operative recovery, the rats were housed in individual boxes with a 12-hour light-dark rhythm (lights on 7.00 am, lights off 7.00 pm). From the BP signal, an algorithm was used to detect systolic arterial pressure (SAP), diastolic arterial pressure (DAP) and pulse interval [22]. Mean arterial pressure (MAP) was calculated on a beat to beat basis and instantaneous HR was calculated from the pulse interval [22]. An index of locomotor activity was obtained by monitoring changes in the received signal strength. For each heartbeat detected, systolic time, SAP, MAP and DAP, pulse interval and locomotor activity were stored in text format on an IBM-compatible computer.

Following an initial 3-day control monitoring period, rats were administered with chronic antihypertensive therapy or vehicle (no treatment) via their drinking water for a period of 2 weeks. The anti-hypertensive agents used were perindopril (1mg/kg/day), or rilmenidine (2mg/kg/day), which we have shown to have antihypertensive properties [20, 23–25]. We determined the concentration by measuring the water intake of the rats for at least 48 hours and adjusting individual water intake and body weight of the rats on a weekly basis. A further 3-day telemetry monitoring period was performed from Day 4–7 and Day 12–14 of the drug treatment period (i.e. day 5, 6, 7, 12, 13, 14). The average over these days was used to determine the effect of each treatment. Ten rats received a single treatment (vehicle n = 4, perindopril n = 3, rilmenidine n = 3), two rats received 2 treatments (vehicle and rilmenidine), while 5 received all three treatments with a fourteen-day recovery period allowed between treatments. Thus there were 17 rats with the total number of treatments being 11, 8 and 10 for vehicle, perindopril and rilmenidine, respectively. The mixture of treatments was designed to include a degree of within animal consistency across the groups with sufficient naïve animals to ensure that the treatments were only somewhat overlapping. The design minimised the use of animals which is desirable from an animal ethics point of view.

### Data analysis

Data for BP, HR and behavioral activity were averaged over 10 minute periods using a specially written analysis and fitting program (CIRCAD version 2003). The data corresponding to each heart beat was initially filtered in order to reject individual data points that exceeded 2.5 times a running standard deviation. The values were then binned into hourly values and fitted to a double logistic curve with the equation:

$$y=P_{1}-P_{2}+\frac{P_{\text{2}}}{1+e^{P_{3}*(x-P_{4})}}+\frac{P_{2}}{1+e^{P_{5}*(x-P_{6})}}$$ (1)

where *P*_*1*_ represented the 'high' plateau, i.e. data obtained during the dark period and *P*_*2*_ represented the data range, i.e. the difference between the calculated 'high' plateau and 'low' plateau. The difference between *P*_*1*_ and *P*_*2*_ then gives the 'low' plateau which indicates data obtained predominantly during the light period. The transition between 'low' and 'high' plateaus was determined as *P*_*3*_ and *P*_*5*_ which are rate of change coefficients. These reflect the steepness of transition from 'high' to 'low' and 'low' to 'high' plateaus, respectively, are independent of the absolute level and can be used to compare different physiological parameters. *P*_*4*_ and *P*_*6*_ are the calculated times at which the respective transitions reach 50%. The method does not assume that the plateau during the day or night is of any particular length and so they can be quite short. In this way the model can cope with data that slowly increase or decrease over several hours or with data that show a particularly short peak or trough which occasionally occurs in some animals.

The program CIRCAD uses an iterative least-squares fitting procedure based upon the Marquardt algorithm [26] with starting parameters for the double logistic fit obtained from an initial Cosinor fitting procedure. Some limitations were set on parameters to ensure sensible final estimates were obtained. The constraints for P_1_ and P2 were established with an initial 'square wave' fit of the data which determined the mean values and standard deviation (SD) for the 'light time' and the 'dark time'. The ‘dark time' mean + 2SD was taken as y max, and the 'light time' mean—2SD was taken as y min. The constraints for P1 and the range P2 were such that the following was true i) y min < = P1 < y max ii) P2 > 0 and iii) y min < = (P1+P2) < y max. Constraints for the curvature parameters P3 and P5 were such that the entire transition between plateaus was at least 30 minutes which meant that at least 10-minute data points must be included in the slope portion. After fitting the 6 parameters of the equation, derived variables were obtained such as slope which is the rate of transition between the 'high' and 'low' plateau. Maximum slope is defined as (P2*P3)/4 for the transition between the 'high' (dark period) plateau and the 'low' (light period) plateau and as (P2*P5)/4 for the transition between the 'low' and 'high' plateau. The formula for the slope is the same as that used for single logistic curve fitting as applied to baroreflex function curves [27].

The power of the transition was calculated as the first derivative of the logistic curve multiplied by the amplitude which is the day night difference between plateaus (Eq 2)

$$\hat{y}=\frac{P2\times P2\times P5\times e^{P5\left(\text{P}6-x\right)}}{{\left(1+e^{P5\left(\text{P}6-x\right)}\right)}^{2}}$$ (2)

The maximum power at the midpoint peak of the curve can be calculated as in Eq 3

$$\hat{y}=\frac{P2\times P2\times P5}{4}$$ (3)

### Cardiovascular variability and cardiac baroreceptor sensitivity

At the end of 2 weeks of treatment, beat-to-beat data from 72-hour recordings were analyzed separately to calculate power spectra using a program written in Labview [28]. The auto- and cross-power spectra were calculated for multiple overlapping (by 50%) segments of MAP and HR using a Fast Fourier transform as adapted for conscious rats [29]. The cardiac baroreflex sensitivity was estimated as the average value of the transfer gain in the mid frequency band (MF) between 0.25 and 0.6 Hz [29, 30]. Baroreflex slope was considered significant if the coherence between MAP and HR across several overlapping segments in the analyzed frequency band was >0.4. Coherence is the frequency domain equivalent of the correlation coefficient and reflects how much of the HR oscillation is due to BP. Data from the light (inactive) period and dark (active) period with low locomotor activity were chosen (4 spectra) from the 72-hour recordings, minimizing the influence of physical activity. Low frequency (LF) MAP power was determined between 0.05 and 0.25 Hz and high frequency (HF) between 0.6 and 1.0 Hz.

### Statistical analysis

Data are presented as mean ± standard error of the mean (S.E.M.) of the between-animal variation. All parameters except P3 and P5 satisfied the normal distribution. For the latter we performed a log transform which normalised the distribution in all cases. Differences between curve parameters for a given physiological measure or between physiological measures for a given curve parameter were compared within groups with a two-way repeated measures analysis of variance (ANOVA) followed by post hoc paired t tests and between group comparisons were made using a one-way ANOVA. Familywise error was accounted for by the Bonferroni procedure. Differences were considered significant when P<0.05.

## Results

### Effect of rilmenidine after 2 weeks of treatment

**Fig 1:**
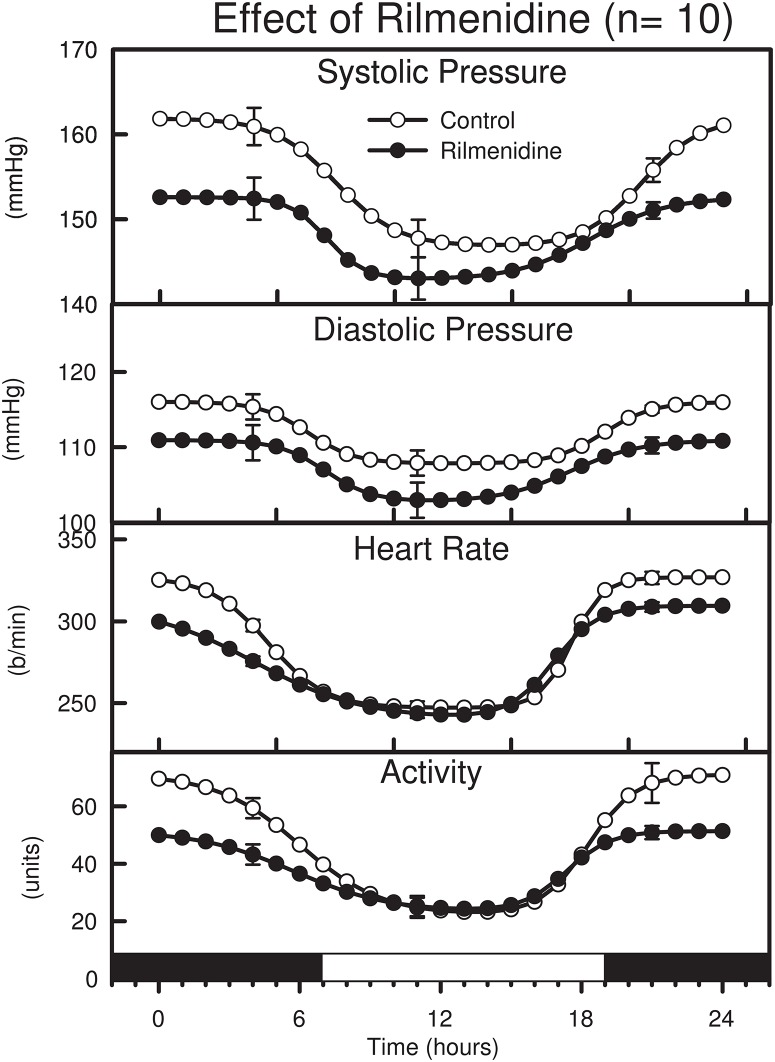
Average double logistic fitted curves showing the circadian variation of systolic and diastolic blood pressure, heart rate and behavioural activity over a 24-hour period from 10 SHR before (open circles) and after (filled circles) treatment with rilmenidine for 2 weeks. Curves were constructed using the average parameters of double-logistic curve fitting procedure. Error bars indicate SEM of hourly averages.

The main effect of chronic treatment with rilmenidine was to reduce SAP, MAP and DAP by -7.5 ± 1.9 mmHg, -7.2 ± 1.7mmHg and -6.2 ± 1.7 mmHg respectively (P<0.01, Table 1, “Fig 1”). The effect on SAP was 2 fold greater during the active period at night compared to the day period (-9.3 ± 2.3mmHg vs -3.9 ± 1.8mmHg, P = 0.003, Table 1, “Fig 1”). There was a smaller effect of rilmenidine on DAP but which was similar during the day and night (-5.1 ± 1.6 mmHg and -5.1 ± 2.0 mmHg respectively, P = 0.01, Table 1, “Fig 1”). There was a reduction in the day night difference in SAP caused by rilmenidine of -5.4 ± 1.7 mmHg (P = 0.002, Table 1, “Fig 1”). Chronic treatment with rilmenidine was less effective at lowering SAP in SHR (P = 0.04) but no difference in the DAP was observed between perindopril and rilmenidine treated SHR (P = 0.09). Chronic treatment with rilmenidine reduced HR and activity during the active period by 5% and 28% respectively (P<0.01) but we did not detect any effect of rilmenidine on these variables during the inactive period (Table 1, “Fig 1”).

**Table 1:** Average results from the circadian analysis of 24-hour blood pressure measurements in rats treated with rilmenidine.

		***Total***			***Diurnal Range***			***Day Plateau***			***Night Plateau***		
*Variable*		*Control*	*Ril*	*P*	*Control*	*Ril*	*P*	*Control*	*Ril*	*P*	*Control*	*Ril*	*P*
**SAP (mmHg)**	Mean	153.9	146.3	***	15.1	9.7	**	146.8	142.9	NS	161.9	152.6	**
	SEM	2.35	2.60		1.38	0.98		2.20	2.49		2.61	3.09	
**MAP (mmHg)**	Mean	132.2	125.0	***	10.8	8.6	NS	127.1	121.6	**	137.9	130.3	**
	SEM	1.96	2.38		0.83	0.92		1.88	2.38		1.92	2.61	
**DAP (mmHg)**	Mean	111.9	105.6	**	8.2	8.2	NS	107.8	102.7	**	116.0	110.9	*
	SEM	1.73	2.31		0.48	1.07		1.67	2.33		1.68	2.36	
**HR (b/min)**	Mean	283.6	270.4	**	79.7	68.8	*	247.1	240.6	NS	326.8	309.5	**
	SEM	3.2	3.3		3.8	2.9		3.6	2.9		4.2	4.6	
**Activity (Units)**	Mean	43.7	37.4	**	48.9	28.0	**	22.2	23.4	NS	71.2	51.4	**
	SEM	3.7	4.2		6.9	2.3		3.5	3.5		7.2	4.4	
		***Dark to Light***			***Light to Dark***			***Dark to Light***			***Light to Dark***		
		***Rate***			***Rate***			***Power***			***Power***		
		*Control*	*Ril*	*P*	*Control*	*Ril*	*P*	Control	Ril	P	Control	Ril	P
**SAP**	Mean	-2.55	-2.80	NS	2.72	1.34	**	-45.94	-38.17	NS	50.54	17.08	**
	SEM	0.39	0.40		0.30	0.23		8.10	8.04		8.61	3.63	
**MAP**	Mean	-2.30	-2.18	NS	2.23	1.18	NS	-29.93	-25.01	NS	30.40	13.94	NS
	SEM	0.31	0.31		0.31	0.24		4.77	4.77		5.90	4.06	
**DAP**	Mean	-1.86	-1.86	NS	1.76	1.24	NS	-18.57	-20.32	NS	18.17	15.63	NS
	SEM	0.24	0.29		0.26	0.23		3.03	3.71		3.39	5.57	
**HR**	Mean	-14.55	-7.62	NS	27.17	16.63	NS	-1164	-585	*	2388	1261	NS
	SEM	3.72	1.21		5.98	1.89		229	78		470	122	
**Activity**	Mean	-6.21	-3.11	NS	10.84	6.74	**	-4.24	-1.04	NS	6.78	2.45	**
	SEM	1.32	0.63		0.86	0.80		1.49	0.22		1.36	0.41	

Absolute values and SEM shown in each column over the 24-hour period.

Significance:

* P <0.05,

** P<0.01,

*** P<0.001, NS P>0.05.

The units of rate are mmHg/hour, b/min/hour or units/hour. The units for power are mmHg^2^/hour, b/min^2^/hour or units^2^/hour. Abbreviations: Ril Rilmenidine, SAP systolic arterial pressure, MAP mean arterial pressure, DAP diastolic arterial pressure, HR heart rate.

Rilmenidine treatment reduced the power of the transition of SAP from light to dark from 50.5 to 17 mmHg^2^/h which is a ~65% reduction (P<0.01) and the rate by ~50% (from 2.7 to 1.3 mmHg/h, P<0.01) but had little effect on the rate of change or power of SAP during the transition from dark to light (Table 1, “Fig 1”). By contrast DAP during either transition was unaffected by rilmenidine. Further, the rate of change and power of the transitions of HR or locomotor activity were differentially affected by rilmenidine. The rate and power of the HR and locomotor reduction during the transition from dark to light and rate and power of the rise during the arousal transition all tended to be less in the presence of rilmenidine but only the HR reduction and the activity power reached statistical significance (Table 1, “Fig 1”).

### Effect of perindopril after 2 weeks of treatment

**Fig 2:**
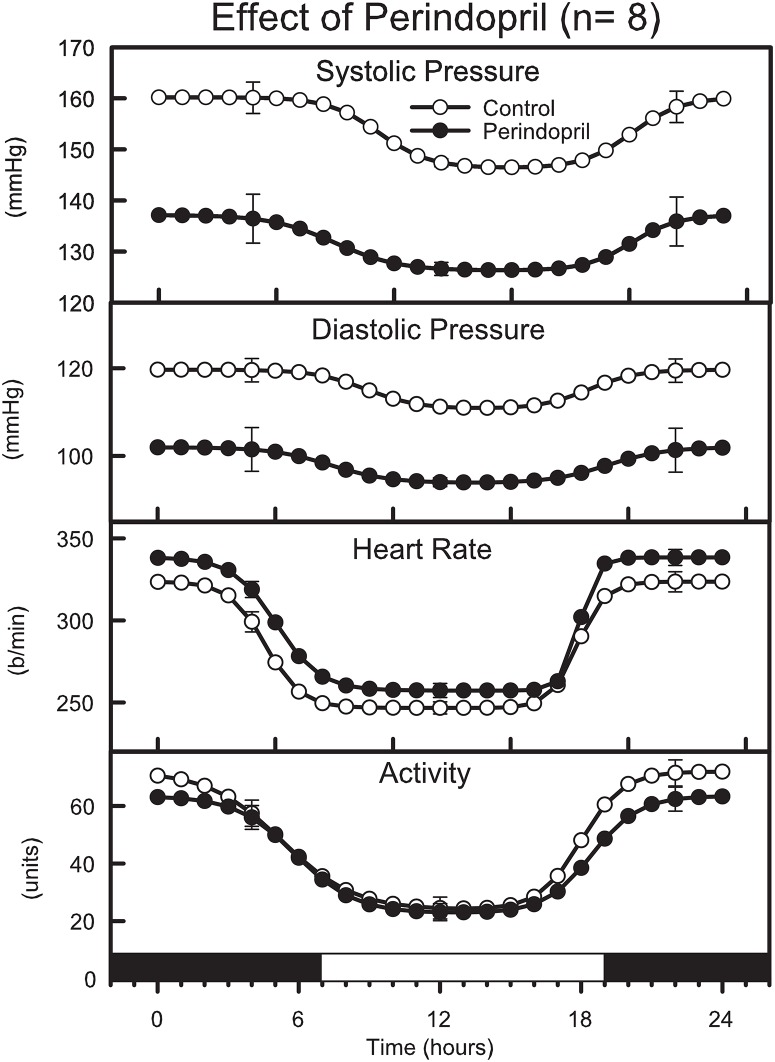
Average double logistic fitted curves showing the circadian variation of systolic and diastolic blood pressure, heart rate and behavioural activity over a 24-hour period from 8 SHR before (open circles) and after (filled circles) treatment with perindopril for 2 weeks. Curves were constructed using the average parameters of double-logistic curve fitting procedure. Error bars indicate SEM of hourly averages.

Chronic administration of perindopril for 2 weeks in the drinking water led to a marked reduction in SAP, MAP and DAP which averaged over 24 hours as 21 ± 3 mmHg, 19 ± 3 mmHg and 16 ± 3mmHg respectively (Table 1, *P*<0.001, “Fig 2”). Furthermore, the reduction in BP was similar during both the night and day period (MAP was 18 ± 4mmHg lower in the day and 19 ± 3mmHg during the night, *P*<0.001). Thus there was no change to the diurnal day night difference (-1 ± 3 mmHg, Table 2, “Fig 2”). Locomotor activity was slightly reduced by perindopril but only during the dark period (-11%, *P* = 0.005, Table 2, “Fig 2”). Perindopril treated SHR had slightly higher HR than at baseline (Table 2, “Fig 2”). Perindopril treatment had no effect on BP, HR or locomotor activity during the transition periods of day to night or night to day since the rate of change and the power of the change was similar in the control and treatment phases (Table 2, “Fig 2”).

**Table 2:** Average results from the circadian analysis of 24-hour blood pressure measurements in rats treated with Perindopril.

		***Total***			***Diurnal Range***			***Day Plateau***			***Night Plateau***		
*Variable*		*Control*	*Perind*	*P*	*Control*	*Perind*	*P*	*Control*	*Perind*	*P*	*Control*	*Perind*	*P*
**SAP (mmHg)**	Mean	154.2	133.1	***	13.8	10.9	NS	146.6	127.8	***	160.4	138.7	***
	SEM	3.67	4.94		0.81	1.34		3.26	4.85		3.56	5.06	
**MAP (mmHg)**	Mean	134.1	115.3	***	10.6	9.2	NS	128.7	110.8	***	139.2	120.0	***
	SEM	2.87	4.68		0.70	1.08		2.86	4.70		2.91	4.67	
**DAP (mmHg)**	Mean	115.1	99.6	***	9.0	8.4	NS	110.9	95.3	***	119.8	103.7	***
	SEM	3.00	4.99		0.79	1.05		2.84	5.08		3.22	4.85	
**HR (b/min)**	Mean	282.7	294.1	*	76.0	82.5	NS	248.6	258.3	NS	324.6	340.8	NS
	SEM	5.9	5.7		3.8	4.4		6.2	5.1		5.2	5.5	
**Activity (Units)**	Mean	47.5	42.5	*	49.4	41.4	NS	24.6	23.4	NS	73.9	64.7	*
	SEM	5.4	4.3		3.8	3.1		4.8	4.4		7.1	5.8	
		***Dark to Light***			***Light to Dark***			***Dark to Light***			***Light to Dark***		
		***Rate***			***Rate***			***Power***			***Power***		
		*Control*	*Perind*	*P*	*Control*	*Perind*	*P*	Control	Perind	P	Control	Perind	P
**SAP**	Mean	-2.82	-1.73	*	2.87	2.54	NS	-45.29	-23.20	*	47.27	41.46	NS
	SEM	0.42	0.31		0.36	0.51		7.67	5.99		6.78	11.17	
**MAP**	Mean	-2.28	-1.56	NS	2.47	2.03	NS	-29.91	-17.37	NS	31.20	27.78	NS
	SEM	0.39	0.27		0.30	0.44		6.37	4.57		4.70	8.51	
**DAP**	Mean	-1.88	-1.54	NS	1.95	1.50	NS	-22.39	-18.04	NS	21.84	16.79	NS
	SEM	0.37	0.33		0.27	0.30		5.79	5.43		4.07	4.68	
**HR**	Mean	-23.57	-22.68	NS	31.18	52.07	NS	-1804	-1989	NS	2613	4811	NS
	SEM	5.87	5.78		7.19	12.18		406	462		574	1142	
**Activity**	Mean	-6.81	-7.44	NS	12.38	9.13	NS	-3.67	-3.90	NS	7.68	4.60	*
	SEM	1.06	1.49		1.31	1.25		0.54	0.91		1.29	0.81	

Absolute values and SEM shown in each column over the 24-hour period. Significance:

* P <0.05,

** P<0.01,

*** P<0.001,

NS P>0.05. The units of rate are mmHg/hour, b/min/hour or units/hour. The units for power are mmHg^2^/hour, b/min^2^/hour or units^2^/hour. Abbreviations: Perind Perindopril, SAP systolic arterial pressure, MAP mean arterial pressure, DAP diastolic arterial pressure, HR heart rate.

### Effect of 2 weeks of vehicle treatment

**Fig 3:**
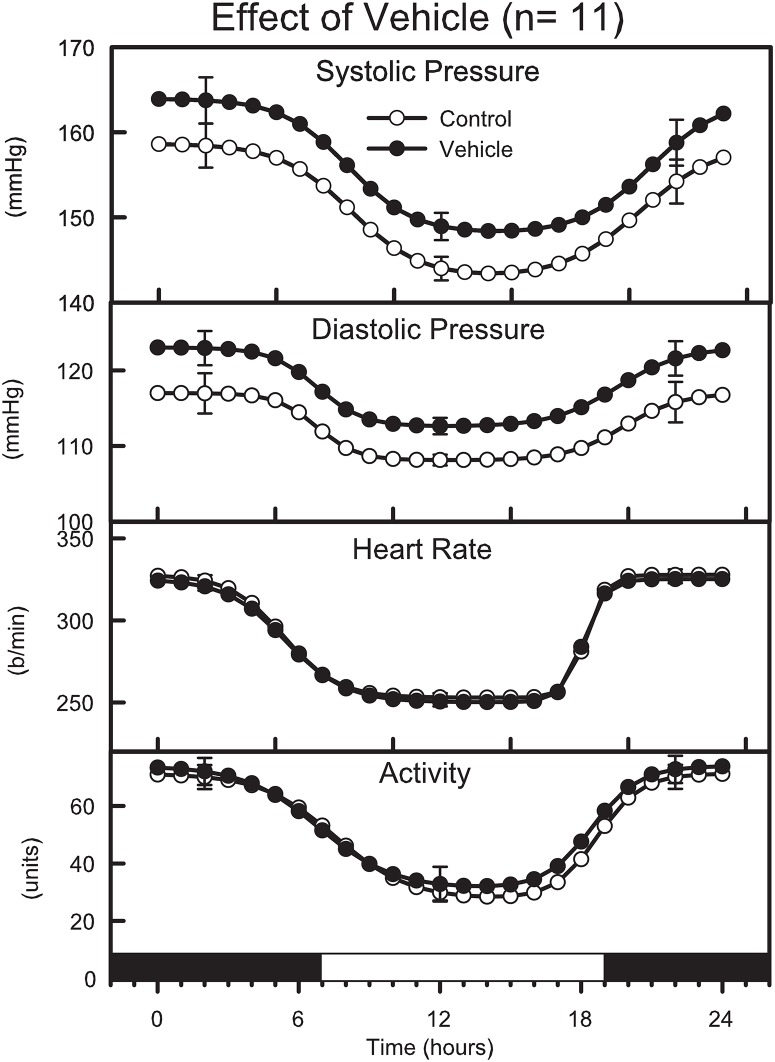
Average double logistic fitted curves showing the circadian variation of systolic and diastolic blood pressure, heart rate and behavioural activity over a 24-hour period from 11 SHR before (open circles) and after (filled circles) treatment with vehicle for 2 weeks. Curves were constructed using the average parameters of double-logistic curve fitting procedure. Error bars indicate SEM of hourly averages.

Vehicle treated SHR at the end of 2 weeks had slightly higher SAP, MAP and DAP than at control (for MAP +5.1 ± 2.0 mmHg, P<0.05, Table 3, “Fig 3”). This effect was evident during both the day and night periods. By contrast HR and activity were closely similar before and after treatment (Table 3, “Fig 3”). There were no differences detected in the day-night range, rate of transition or power for any variable (Table 3, “Fig 3”).

**Table 3:** Average results from the circadian analysis of 24-hour blood pressure measurements in rats treated with Vehicle.

		***Total***			***Diurnal Range***			***Day Plateau***			***Night Plateau***		
*Variable*		*Control*	*Vehicle*	*P*	*Control*	*Vehicle*	*P*	*Control*	*Vehicle*	*P*	*Control*	*Vehicle*	*P*
**SAP (mmHg)**	Mean	150.4	155.2	*	15.9	15.9	NS	142.8	148.0	*	158.7	163.9	*
	SEM	2.40	2.61		1.38	1.60		2.58	2.70		2.74	2.80	
**MAP (mmHg)**	Mean	130.8	135.9	*	10.9	12.2	NS	125.6	130.1	*	136.5	142.3	*
	SEM	2.20	2.11		0.73	1.43		2.37	2.24		2.32	2.18	
**DAP (mmHg)**	Mean	112.2	117.6	*	8.9	10.5	NS	108.1	112.6	*	117.0	123.0	*
	SEM	2.68	2.28		0.71	1.08		2.68	2.28		2.77	2.41	
**HR (b/min)**	Mean	287.5	285.6	NS	74.8	75.0	NS	252.9	250.1	NS	327.7	325.1	NS
	SEM	3.1	2.6		2.5	2.9		3.4	2.5		3.3	3.1	
**Activity (Units)**	Mean	50.5	51.4	NS	43.7	42.6	NS	27.5	31.2	*	71.1	73.8	NS
	SEM	4.2	4.3		2.8	6.0		4.2	4.7		5.2	6.5	
		***Dark to Light***			***Light to Dark***			***Dark to Light***			***Light to Dark***		
		***Rate***			***Rate***			***Power***			***Power***		
		*Control*	*Vehicle*	*P*	*Control*	*Vehicle*	*P*	Control	Vehicle	P	Control	Vehicle	P
**SAP**	Mean	-2.35	-2.51	NS	2.12	2.36	NS	-45.04	-48.13	NS	42.06	48.45	NS
	SEM	0.32	0.34		0.22	0.26		7.51	7.66		6.67	9.68	
**MAP**	Mean	-2.76	-2.75	NS	1.94	2.16	NS	-38.50	-40.28	NS	25.67	33.74	NS
	SEM	0.37	0.30		0.22	0.25		6.85	6.30		3.55	7.16	
**DAP**	Mean	-2.35	-2.39	NS	1.64	1.70	NS	-27.86	-31.68	NS	18.23	23.03	NS
	SEM	0.30	0.27		0.20	0.20		5.36	5.28		2.69	4.28	
**HR**	Mean	-14.69	-15.11	NS	40.75	36.51	NS	-1162	-1191	NS	3431	2993	NS
	SEM	2.79	2.57		7.31	5.84		191	169		642	440	
**Activity**	Mean	-6.18	-5.95	NS	10.55	9.59	NS	-3.01	-3.12	NS	5.71	5.39	NS
	SEM	0.91	0.75		1.07	0.84		0.46	0.87		0.83	1.22	

Absolute values and SEM shown in each column over the 24-hour period. Significance:

* P <0.05,

** P<0.01,

*** P<0.001,

NS P>0.05. The units of rate are mmHg/hour, b/min/hour or units/hour. The units for power are mmHg^2^/hour, b/min^2^/hour or units^2^/hour. Abbreviations: SAP systolic arterial pressure, MAP mean arterial pressure, DAP diastolic arterial pressure, HR heart rate.

### Effect of treatments on arousal surge power function curves of SAP

**Fig 4:**
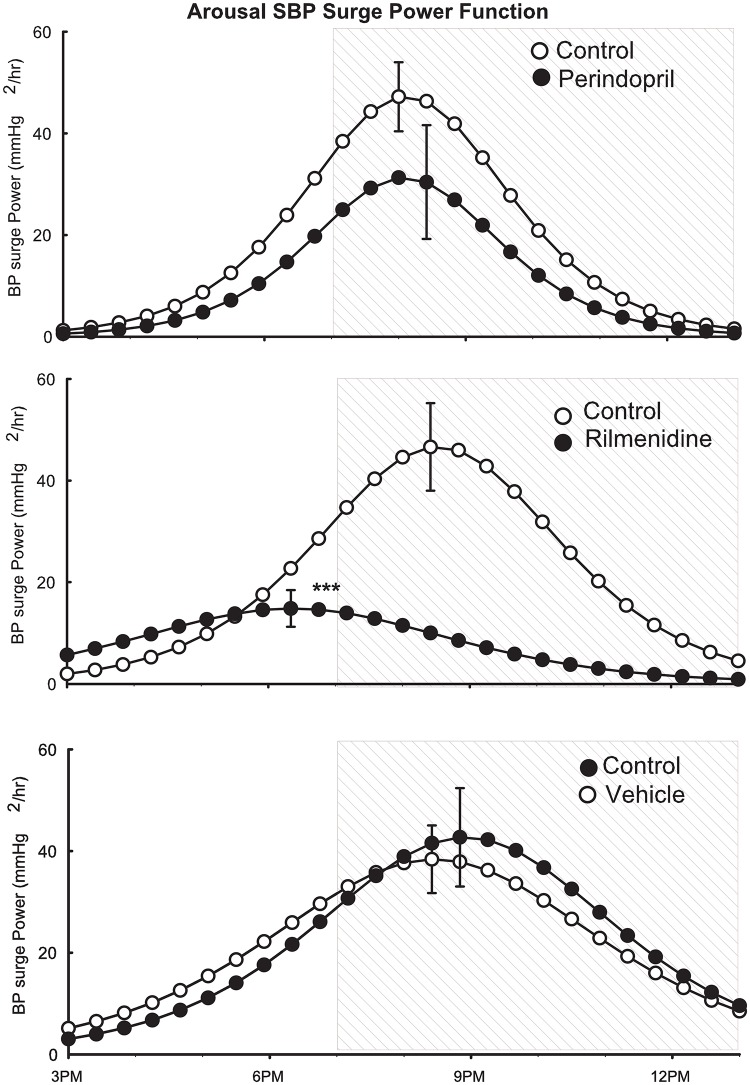
Average double logistic fitted curves showing the circadian variation of systolic and diastolic blood pressure, heart rate and behavioural activity over a 24-hour period from 11 SHR before (open circles) and after (filled circles) treatment with vehicle for 2 weeks. Curves were constructed using the average parameters of double-logistic curve fitting procedure. Error bars indicate SEM of hourly averages.

During the onset of the dark active period, the power of the surge can be plotted using the average calculated variables for the derivative of the logistic equation. The peak of the power surge occurs 2–3 hours after the onset of the dark (lights off) (“Fig 4”). The maximum and the timing of the peak were not affected by perindopril or by vehicle. However, rilmenidine markedly attenuated the maximum of the curve such that it occurred 2–3 hours before the onset of darkness (“Fig 4”).

### Blood pressure variability

**Fig 5:**
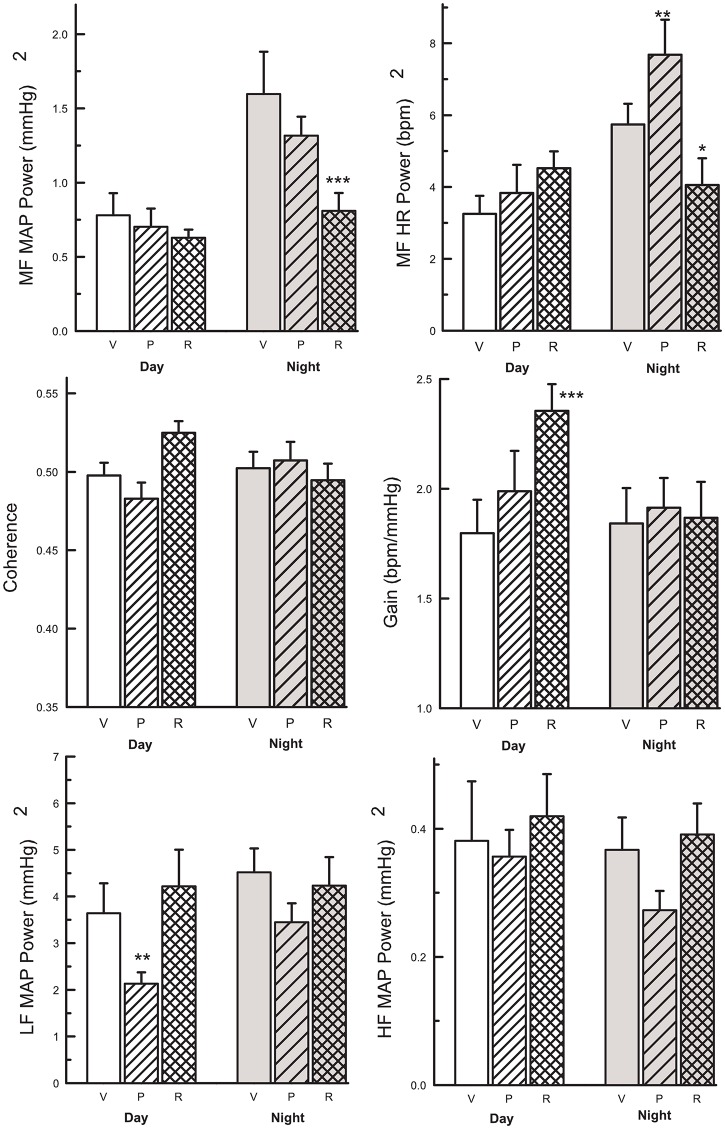
Upper: Average mid frequency (MF, 0.25–0.6 Hz) MAP power (mmHg)^2^, MF HR power (bpm)^2^, Middle: Coherence (units), baroreflex gain, (gain, bpm/mmHg), Lower: low frequency (LF, 0.05–0.25 Hz) and high frequency (HF, 0.6–1.0 Hz) MAP power (mmHg)^2^ from cross spectral analysis during the light (inactive) phase (left bars white), and dark (active) phase (right bars, shaded). Measurements are 2 weeks after vehicle (V, open bars), perindopril (P, hatched bars) and rilmenidine (R, crosshatched bars) in SHR (n = 7–9 per group). Values are mean ± SEM. Effect of perindopril or rilmenidine compared with vehicle *, P<0.05; **, P<0.01; ***, P<0.001.

During the dark (active) period, MF MAP power was 105% greater in vehicle treated SHR (*P*<0.001) and 87% greater in perindopril treated SHR (*P*<0.001) than that observed during the day inactive period. However, we did not observe a day night difference in rilmenidine treated SHR (+29%, P = 0.7, “Fig 5”). Thus while no treatments affected the MF MAP power in the day inactive period, rilmenidine reduced the power to similar levels as that observed during the inactive period (“Fig 5”). This effect was still evident if the power was normalised against the change in basal MAP (-40%, P<0.001, “Fig 5”).

During the dark (active) period, LF and HF MAP power in vehicle treated SHR were similar to those recorded during the inactive period (“Fig 5”). Rilmenidine treated rats had similar daytime and night-time LF and HF power as that recorded from vehicle treated SHR. In contrast perindopril treated SHR had lower LF power than vehicle treated SHR during the day (P<0.01) and a trend for LF power to be less during the night period. However, when the power was normalised for the basal level of MAP there was no effect of perindopril (“Fig 5”). There were no differences between groups during the day or night in the HF MAP band (“Fig 5”).

### Heart rate variability and cardiac baroreflex sensitivity

MF HR power was 76% greater in the dark active period compared to the day inactive period in vehicle treated SHR (P = 0.001) but baroreflex gain was similar in the day and night periods (“Fig 5”). Perindopril treatment did not alter MF HR power during the inactive period (day) but increased HR MF power during the active period (P<0.01, “Fig 5”). However, there were no effects of perindopril on baroreflex gain. By contrast, rilmenidine treated SHR had greater baroreflex gain (+31%, P<0.001) during the inactive period compared to vehicle (“Fig 5”). The coherence between MAP and HR at the MF band was similar during all treatments and phases of the cycle.

### Recovery of blood pressure post experiment

The duration of the complete experiments ranged from 5–14 weeks. The aging of animals during this time had no effect on MAP in that the initial control values did not differ from those observed during the recovery from treatment period (127 ± 2 mmHg vs 130 ± 4 mmHg, P = 0.35).

## Discussion

In this study we have shown that chronic treatment with rilmenidine and perindopril reduced BP in SHR but rilmenidine was the only treatment to very strongly limit the arousal induced surge in BP that occurs with the onset of the dark period when the rats become active. Presumably the greater effect of rilmenidine in the active period reflects the greater sympathetic vasomotor drive during this time as is reflected in the selective reduction in the MF power. The MF has been shown to be related to sympathetic drive in mice [31] and rabbits [32]. Rilmenidine had no effect on variability of BP in the LF or HF bands which are not thought to be related to sympathetic activity [32]. Chronic treatment with perindopril was more effective at lowering BP in SHR but had no detectable effect on the arousal surge in BP with the onset of the dark period nor on the MF (autonomic) power. Perindopril did reduce power in the LF band during the day and there was a trend to reduce variability in the LF and HF bands during the active night period. However, these effects disappeared when the change in MAP was accounted for. This suggests that the effect of reducing variability may be related to the reduction in MAP itself. Previous studies in normotensive rats have not shown any effects on BP variability measured by standard deviation or by co-efficient of variation by an acute dose of perindopril [33]. These measures include all frequency bands. However, the acute dosing regime did not alter BP unlike our current study with chronic treatment. This further suggests that the observed reduction in LF with perindopril is secondary to the change in BP. We have previously shown that captopril which is another angiotensin converting enzyme inhibitor, reduced myogenic LF and very LF tubule-glomerular feedback in renal vascular conductance suggesting that this might be the mechanism [32]. In that study in rabbits, rilmenidine had no effect on these LF mechanisms and was confined to reducing the frequencies associated with sympathetic fluctuations. This is consistent with the current findings where the effect of rilmenidine in reducing MAP power in the active period was confined to the autonomic band and had no effect on the HF or LF bands when compared to vehicle. Also rilmenidine selectively increased LF but not MF or HF during the inactive day period. The effects of rilmenidine persisted after normalising for changes in basal MAP. These findings support the view that the arousal surge in BP is very heavily dependent on activation of the SNS in SHR.

One of the interesting and perhaps unexpected finding was that the attenuation of the rate and power of the arousal surge in BP by rilmenidine was only observed in SAP and not in DAP. The rate and power of the rise in BP reflect the activation of the SNS and changes in a range of other hormones such as increasing catecholamine plasma concentrations, glucocorticoids such as cortisol (in humans) and components of the renin angiotensin system [34]. Importantly, the morning surge in the rate and power of the rise in humans is greatest in hypertensive patients [1, 2] and is an independent predictor of cardiovascular events [35]. Presumably the rate of fall and the power of the fall is related to the opposite changes as one approaches sleep. In SHR the rate and amplitude of the arousal surge in SAP is 47% and 53% greater than that for DAP and thus the power being the multiplicand of these is 2.2 times greater for SAP than DAP. During rilmenidine treatment the power of the surge in SAP was similar to that for DAP so the main effect of rilmenidine was quite selective for the SAP over DAP. Previous studies such as those by Janssen that have examined the timed effect of administering rilmenidine to conscious SHR have only reported MAP and not SAP or DAP. Further, Janssen used a bolus of rilmenidine just three hours before lights are turned off rather than a steady state infusion of the drug [24]. Thus these methodological differences make it difficult to compare with the findings of the current study. The study by Monassier and colleagues examined continuous subcutaneous administration with rilmenidine given by minipump to SHR and showed similar reductions in SAP and DAP as the present study if one compares the vehicle with rilmenidine at the end of the study [36]. In this study there was no assessment of the rate or power of the change from light to day. Thus our study is novel for this type of analysis in SHR. We have previously reported that the rate of rise in MAP in hypertensive BPH/2J mice and normotensive control strain BPN/3J was largely attenuated by administration of rilmenidine via minipump [20]. The extent of the BP lowering by rilmenidine was also similar to the present study but we did not report on whether the effect was observed in SAP or DAP [20]. We did observe a nearly 80% reduction in the MAP surge during the onset of the dark period which clearly must involve both SAP and DAP. Indeed, a closer look at the analysis reveals both SAP and DAP were similarly affected in the BPH/2J mice (unpublished observations) suggesting a difference between this strain of mouse and the SHR where only the SAP surge was affected. There are a number of studies that have shown that rilmenidine equally reduces SBP and DBP in humans [37] during the day. Perhaps the most relevant is the study in which Finta and colleagues examined the effect of rilmenidine on the circadian pattern of SBP and DBP. They found no effect on either SBP or DBP during the night but a large effect during the day with the effect on SBP being nearly double the effect in DBP. Further analysis of their data revealed that the biggest morning step rise per hour in the morning was reduced by 70% for SBP and only 44% for DBP [38].

One possibility is that the nocturnal pattern of SAP changes in SHR are mediated more by the SNS but not those involving the DAP changes. This is unlike the BPH/2J mice where the SNS contributes equally to both measures of BP. Doxazosin, an alpha-adrenoreceptor antagonist, is most effective in reducing morning hypertension particularly if administered at bedtime [39]. Similarly, the centrally acting sympatho-lytic agents guanabenz or clonidine are most effective at reducing the morning hypertension in patients also when given at bedtime [40]. Other studies in humans using ambulatory BP recordings have included analysis as to whether treatments can selectively target the morning surge in blood pressure or at least the morning hypertension. Chronotherapy with the slow release calcium channel blocker nifedipine reduced the morning surge but only after evening administration and did not affect the awake to sleep ratio in SAP or DAP [41]. However, the calculation of the morning surge as the BP 2 hours after waking minus the lowest BP during the night is a measure that is unclear what exactly it reflects. Our cross-sectional analysis however, did show that patients taking calcium channel blockers had a reduced morning surge power in BP as did diuretics while ACE inhibitors had no effect [1]. The latter is consistent with the current study using perindopril [1].

One of the strengths of the current study was to include another type of antihypertensive agent that is very effective in lowering BP in SHR but that doesn’t appear to affect the sympathetic nervous system, namely perindopril [42]. The dose of perindopril of 1 mg/kg/day has been used previously in SHR [43] and in rabbits and is in the middle of the dose response curve [43, 44]. In the present study we observed no effect of perindopril on the rate or power of the arousal associated rise in BP. However, we did observe a reduction in variability in some frequency bands particularly the LF band. If we express the power as a percentage of the absolute level of BP then perindopril has no effect. This suggests that this is related to the reduction in BP overall and not to an inhibition of the sympathetic nervous system. Perindopril is known to reduce BP variability in sympathectomised rats but not in normotensive rats [33]. In the latter case perindopril was given acutely and did not reduce BP. Thus it is likely that perindopril has no discernible effect on the sympathetic nervous system in our current study. The lack of effect on the arousal surge is consistent with our analysis of ambulatory BP recordings in humans where we found that those hypertensive patients taking angiotensin converting enzyme inhibitors or angiotensin receptor blocking drugs had a similar rate and power of their morning surge in BP as untreated hypertensive patients [35]. This was a cross-sectional study so the conclusions are only speculative at this stage and would need to be confirmed in a blinded placebo controlled cross over study.

In conclusion the present study suggests that the arousal associated with hypertension can be specifically targeted by a centrally acting sympatholytic agent such as rilmenidine at doses that produce a modest hypotension. This contrasts angiotensin converting enzyme inhibitors which are very effective antihypertensive agents but do not alter the circadian pattern of BP variability. These studies suggest that centrally acting drugs may be ideal to counter the cardiovascular risk associated with the morning surge in patients. Clearly only a long term outcome study would be able to determine the effectiveness of this strategy.

## Supporting Information

S1 File Supporting File. (XLSX)
